# Proportional Trends in Pediatric Opioid Prescribing Between 2005 and 2016 by Age Group, Sex, Ethnicity, Race, Language, and Payer Status from a Large Children’s Hospital in the Southwest United States

**DOI:** 10.3390/children11111356

**Published:** 2024-11-08

**Authors:** Melissa Pielech, Eric Kruger, Samantha M. Portis, Khirsten J. Wilson, W. Evan Rivers, Kevin E. Vowles

**Affiliations:** 1 Department of Psychiatry and Human Behavior, Warren Alpert Medical School of Brown University, Providence, RI 02903, USA; melissa_pielech@brown.edu; 2 Department of Behavioral and Social Sciences, Center for Alcohol and Addiction Studies, Brown University School of Public Health, Providence, RI 02903, USA; 3 Division of Physical Therapy, Department of Orthopedics and Rehabilitation, School of Medicine, University of New Mexico Health Sciences Center, Albuquerque, NM 87131, USA; 4 Department of Psychological Sciences, University of Missouri, Columbia, MO 65211, USA; 5 Department of Psychology, University of Notre Dame, Notre Dame, IN 46556, USA; 6 Physical Medicine and Rehabilitation Service, Tennessee Valley Healthcare System, Department of Veterans Affairs, Nashville, TN 37212, USA; 7 Department of Physical Medicine and Rehabilitation, Vanderbilt University Medical Center, Nashville, TN 37232, USA; 8 School of Psychology, Queen’s University Belfast and Belfast Centre for Pain Rehabilitation, Belfast City Hospital, Belfast BT9 7AB, UK

**Keywords:** opioids, prescribing trends, pediatrics, children, age-related differences

## Abstract

Background/Objectives: Prescription opioid use before adulthood is typically effective for acute pain control and is also associated with adverse short- and long-term consequences. Methods: This study examined pediatric opioid prescribing trends over time across different age groups (early childhood, school age, adolescence, young adult) and sociodemographic subgroups (sex, ethnicity, race, language, payer type) from 2005 to 2016. Results: Utilizing 42,020 first outpatient opioid prescriptions for youth aged 0–21 years from a large US children’s hospital, this research found notable trends and disparities. Prescription rates increased by 35% from 2005–2007 to 2008–2010, then decreased by 14% from 2008–2010 to 2011–2013, and decreased again by 22% from 2011–2013 to 2014–2016. Chi-squared tests indicated significant changes in prescription rates across all sociodemographic subgroups, though only age group, ethnicity, and payer type (i.e., the party responsible for payment for hospital services) had changes with non-negligible effect sizes (Cramer’s V). Specifically, age group showed small to medium effects (V = 0.16), while ethnicity and payer demonstrated small effects (V = 0.10 each). This study highlights variations in opioid prescribing trends, particularly among different age groups, ethnicities, and payer statuses up to 2016. Conclusions: These findings reveal differing trends in pediatric opioid prescribing during the peak of the opioid epidemic, highlighting the importance of considering age and sociodemographic variables for understanding prescribing patterns fully and raising potential concerns about inequities in pain management. Future studies should explore similar trends from 2016 onward.

## 1. Introduction

Opioid prescription during childhood and adolescence may increase the short- and long-term risk of opioid-related adverse consequences, including opioid misuse, overdose, and substance use disorders (e.g., [1,2,3]). An estimated 2.3% of adolescents and 5.2% of young adults report using prescription pain relievers differently than prescribed (i.e., “opioid misuse”), with their own or a relative/friend’s prescription identified as the source [4]. Past-month receipt of an opioid prescription has been associated with an over seven-fold increase in overdose risk in adolescents and young adults [5]. Using prescribed opioids before the end of high school is associated with a 33% increase in risk for future opioid misuse [6].

Although recent pediatric opioid prescribing rates are trending downward (e.g., [7,8]), opioid prescribing to US youth increased over the previous two decades, and opioids are still commonly prescribed to pediatric patients (see ref. [9] for an in-depth review of studies on pediatric opioid prescribing trends published through 2018). Several sociodemographic (i.e., age) and health-related individual characteristics (i.e., multiple pain complaints) in youth have been associated with the likelihood of receiving an opioid prescription, yet studies examining longitudinal prescribing trends within sociodemographic subgroups of patients are limited. Rather, opioid prescription trends among youth are often reported in aggregate across ages or within one age group (i.e., adolescence). This approach fails to account for differences present in the developmental spectrum, including how contextual factors may influence risks and outcomes associated with prescription opioids. To our knowledge, only two studies have examined pediatric opioid prescription trends over time within different age groups. Renny and colleagues [7] examined temporal opioid dispensing trends for children, adolescents, and young adults from 2006 to 2018 and found decreases in prescriptions of long-duration and high-dosage opioids among adolescents and young adults, yet there were increases in the prescribing of long-duration opioids for youth below age 10. Groenewald and colleagues also examined prescribing trends over time between age subgroups but did not find notable differences between groups [10].

The consideration of prescribing trends over time within other sociodemographic subgroups (e.g., race, ethnicity, sex) may also yield helpful information for understanding pain treatment practices, outcomes, and disparities among diverse youth. Groenewald and colleagues quantified the national rates of prescriptions for opioids and non-opioid analgesics over time by race and ethnicity [11]. Within this sample, there were higher opioid prescribing rates to White youth than Black, Asian, or Hispanic children and higher prescribing of non-opioid analgesics to youth from racially diverse groups [11]. Consistent with the *JAMA* guidelines on reporting on race and ethnicity, we interpret race and ethnicity as social constructs that reflect a complex relationship with ancestry, heritage, social determinants of health, and socioeconomic, structural, institutional, cultural, and demographic factors [12].

The present study is a secondary analysis of electronic medical record data on outpatient opioid prescriptions to youth within the primary hospital system in New Mexico during the peak of opioid prescribing in the US (2005–2016). Among other findings, the primary study [13] found an increased risk of adverse outcomes in patients who were 18–21 years old and that patients from diverse racial and ethnic backgrounds were less likely to receive multiple opioid prescriptions but more likely to experience adverse outcomes. The primary aim of this study was to examine changes in the proportion of prescriptions by age group at the time of prescription (early childhood, school age, adolescence, young adult) and other sociodemographic subgroups, specifically sex, ethnicity, race, language, and payer status. This study was exploratory and thus did not have prespecified hypotheses with regard to the magnitude or type of proportional change in prescriptions by subgroup.

## 2. Materials and Methods

### 2.1. Data Source

Data were retrieved from the University of New Mexico Hospital electronic medical record system between 1 January 2005 and 21 December 2016 and fully deidentified by the University of New Mexico Health Sciences Center. Eligible patients were between the ages of 0 and 21 years at the time of prescription and received an opioid prescription during an outpatient clinical encounter (e.g., outpatient clinic, emergency department visit, discharge from inpatient stay, day surgery; see ref. [13] for additional details). Data on inpatient opioid prescriptions were excluded. Specifically, data were anonymized during extraction via an honest broker, and a unique ID number was assigned to each individual in the dataset in order to link information from multiple clinical encounters. Institutional review board approval was obtained for data extraction and analysis.

### 2.2. Sample

The sample included all patients (aged 0 to 21) who received an outpatient opioid prescription (i.e., emergency department, discharge from inpatient admission, day surgery or outpatient clinic) between 2005 and 2016. Inpatient opioid prescriptions were excluded; however, prescriptions administered at discharge from an inpatient stay were included. Prescriptions that could be used for medication treatment of an opioid use disorder (MOUD, e.g., methadone or buprenorphine) were excluded. To control for the results being inflated if multiple prescriptions were written for a patient, only the first opioid prescription received by a patient during the study time period was included in the analyses (*N* = 42,020).

Relevant baseline medical and sociodemographic factors extracted for each patient included age at first prescription encounter (during the study time frame), biological sex, race, ethnicity, and primary language. Both race and ethnicity were originally self-report items that patients or their parents self-reported during patient registration procedures according to the forms used by the healthcare organization. We acknowledge that both race and ethnicity are complex social constructs that are correlated with other sociodemographic variables, for example, socioeconomic status, discrimination, healthcare access, and patient outcomes. We are using these variables to illustrate potential disparities between groups; however, we acknowledge that the specific causes of these disparities cannot be determined by the data. Patient age at the first opioid prescription was categorized as early childhood (0–5 years), school age (6–11 years), adolescence (12–17 years), or young adult (18–21 years). Racial categories were American Indian/Alaskan Native, Asian, Black or African American, Hawaiian Native or Pacific Islander, and White. Ethnicity was classified separately from race and included two categories: “Hispanic/Latino” or “Not Hispanic/Latino”. The electronic health record also contained responses to racial categories including “decline to answer”, “two or more”, and “other”. These responses were excluded from analysis because of their low counts and their unclear conceptual meaning. Payer type was one of three categories (private insurance, public (i.e., Medicaid), or uninsured) and represents the party responsible for the majority of the payment of healthcare services.

The frequencies of opioid prescriptions were summed and tabulated into four three-year periods based on years of prescriptions, 2005–2007, 2008–2010, 2011–2013, and 2014–2016, for three reasons. First, there were low counts of race categories in some of the individual years. Second, the three-year periods corresponded to trends of the data that were originally reported by Pielech et al. (2020) [13]. Third, we wanted to increase the stability of the estimates of opioid prescriptions across time; that is, we wanted to minimize the effect that year-to-year variability in opioid prescriptions would have on the chi-square test (see below).

### 2.3. Statistical Analyses

Database merging, data cleaning, data coding, and statistical analysis were conducted using R version 4.3.0. [11]. A primary challenge in analyzing these data was the limited contextual information obtained from each patient visit. In more specific terms, in the EHR data we had access to, we could not determine whether and why a pain medication (such as an opioid) was indicated and, if it was indicated, whether it was prescribed as the primary pain medication or co-prescribed with an additional pain medication (e.g., nonsteroidal anti-inflammatory drug). Stated differently, we did not have data on all encounters where a pain medication (of any kind) or another pain treatment would be indicated. Thus, we could not determine whether opioids were more frequently prescribed in encounters out of the total possible encounters where opioids could be prescribed.

Since we did not have that data, we elected to only calculate the proportional change in the numerator, that is, the relative change in opioid prescriptions over time per patient characteristic (i.e., age group, sex, ethnicity, race, language, and payer status). Given that we had complete data from an entire health system, we elected to use chi-square tests to determine proportional change over time, which does not require a denominator to determine whether proportions change over time. Proportional changes were evaluated between periods of prescriptions; sociodemographic subgroups (age, sex, payer, race, ethnicity, and language) were evaluated by Pearson chi-squared tests, and effect sizes were calculated via Cramer’s V statistic [14]. Statistical significance tests, particularly the chi-squared test, are sensitive to large sample sizes; therefore, the effect size (Cramer’s V) was evaluated to determine whether associations were worthy of consideration [15]. Cohen’s guidelines for effect sizes were used to determine negligible, small, medium, and large effect sizes [15]. Effect sizes larger than a negligible association were considered a meaningful effect. 

## 3. Results

**Figure 1:**
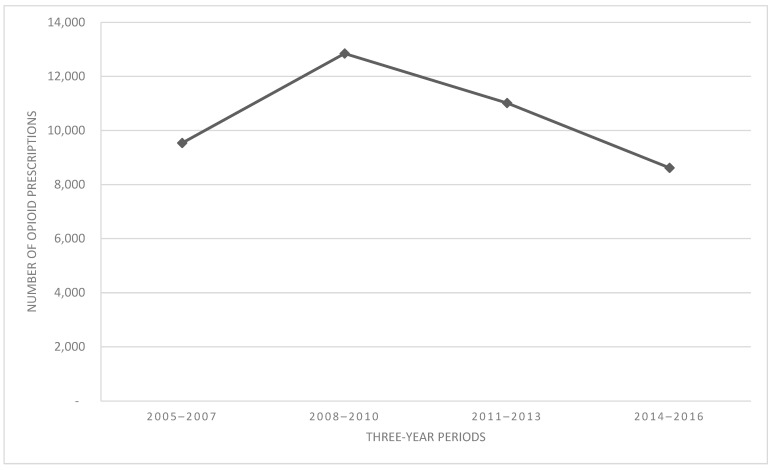
Overall trends over time. Note: Trends in absolute opioid prescription with opioid prescriptions aggregated into three-year periods.

A total of 40,020 first-time opioid prescriptions were identified in patients (aged 0–21 years) from 2005 to 2016. Age at the time of the first opioid prescription ranged from 0 to 21 years (M = 13.5, SD = 6.50), and just over half the sample (55.0%) was male. Please refer to Table 1 for reporting of opioid prescriptions by sociodemographic subgroups and refer to the primary outcomes paper [13] for additional prescription-related characteristics. Trends for total opioid prescriptions by three-year period are reported in Figure 1. Prescriptions increased by 35% from the 2005–2007 to 2008–2010 periods and then demonstrated a steady decrease of −14% from 2008–2010 to 2011–2013 and then a further decrease of −22% from 2011–2013 to 2014–2016.

### 3.1. Prescribing Trends by Age Group at Time of First Prescription

**Figure 2:**
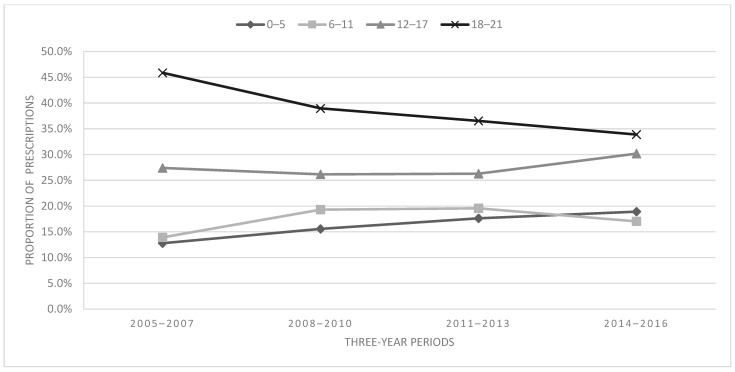
Proportion of prescriptions by age group.

Figure 2 depicts the proportion of prescriptions by age group aggregated into three-year periods. Across all periods, the young-adult-aged patients made up the largest portion of the sample (38.9%), followed by adolescents (27.3%), while school-age and early-childhood patients accounted for 16.2% and 17.7% of the sample, respectively. The association between age and time was significant [χ^2^ = 471.75, df = 9, *p* < 0.001, V = 0.06 (small effect)]. The percentage increase in the proportion of prescriptions for young adults decreased by 26.1% from 2005–2007 to 2014–2016, while prescriptions increased for adolescents (10.2%), school age (22.2%), and early childhood (47.9%).

### 3.2. Prescribing Trends by Sex

**Figure 3:**
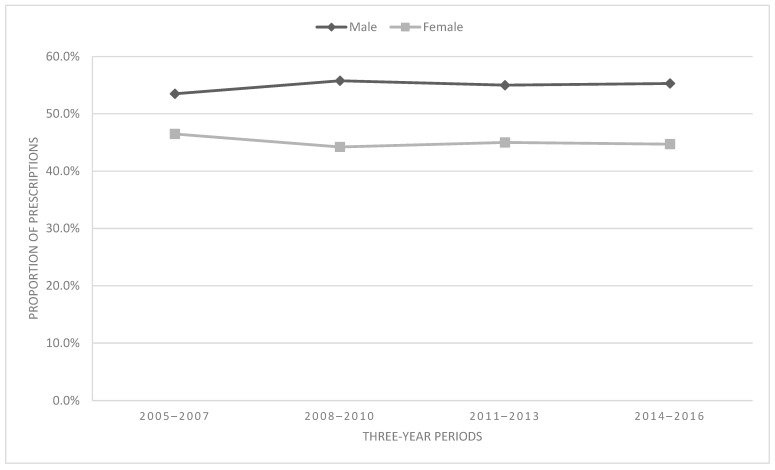
Proportion of prescriptions by sex.

Overall, more males (55.0%) than females (45.0%) received opioid prescriptions. The association between sex and time was significant [χ^2^ = 11.88, df = 3, *p* = 0.008, V = 0.016 (negligible effect)]. The proportion of males receiving prescriptions increased by 3.3% from 2005–2007 to 2014–2016 and decreased by 3.8% for females during the same period. Figure 3 displays trends based on sex by time period.

### 3.3. Prescribing Trends by Ethnicity

**Figure 4:**
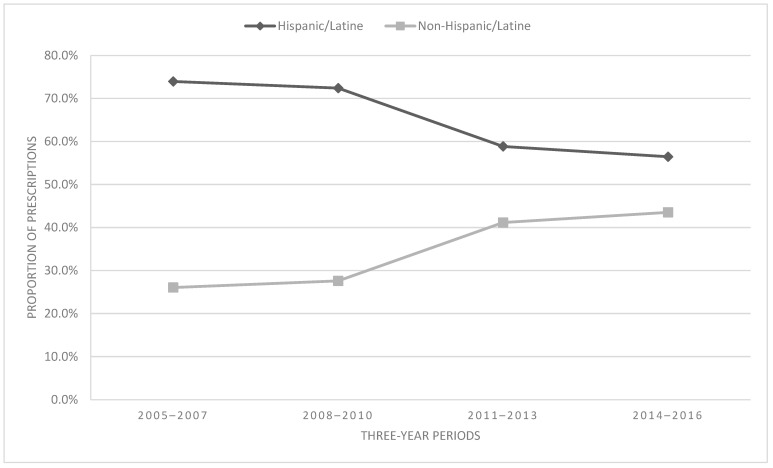
Proportion of prescriptions by ethnicity.

Regarding prescribing trends among ethnic groups, see Figure 4. The majority of prescriptions were to Hispanic/Latine patients (64.7%). Ethnicity and time were significantly associated [χ^2^ = 836.93, df = 3, *p* < 0.001, V = 0.16 (small/medium effect)]. Hispanic/Latine patients demonstrated a 23.6% decrease in opioid prescriptions from 2005–2007 to 2014–2016, while non-Hispanic/Latine patients simultaneously demonstrated a 67.1% increase in prescriptions during this same period.

### 3.4. Prescribing Trends by Race

**Figure 5:**
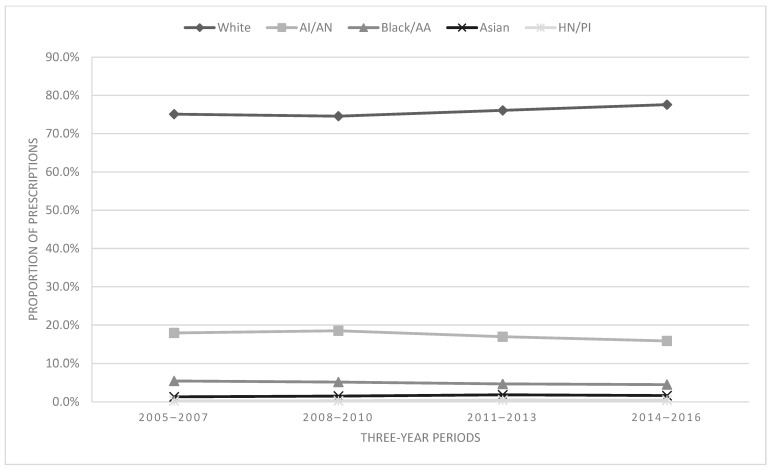
Proportion of prescriptions by race.

In total, across the study time frame, 75.9% of the prescriptions went to White individuals, 17.3% to American Indiana/Alaskan Native, 4.9% to Black/African American, 1.6% to Asian, and 0.4% to Hawaiian Native/Pacific Islander (see Figure 5). Racial subgroups and time were significantly associated [χ^2^ = 41.51, df = 12, *p* < 0.001, V = 0.02 (negligible effect size)]. There was a minimal proportional change for White individuals (3.3%) from 2005–2007 to 2013–2016, while American Indian/Alaskan Native (−11.6%) and Black/African American (−17.3%) individuals both experienced decreases. At the same time, Asian (24.1%) and Hawaiian Native/Pacific Islander (91.7%) individuals experienced the largest relative increases in the proportion of opioid prescriptions (see Figure 5). However, the changes in the proportion of prescriptions in these two groups should be interpreted cautiously due to relatively small sample sizes.

### 3.5. Prescribing Trends by Preferred Language

**Figure 6:**
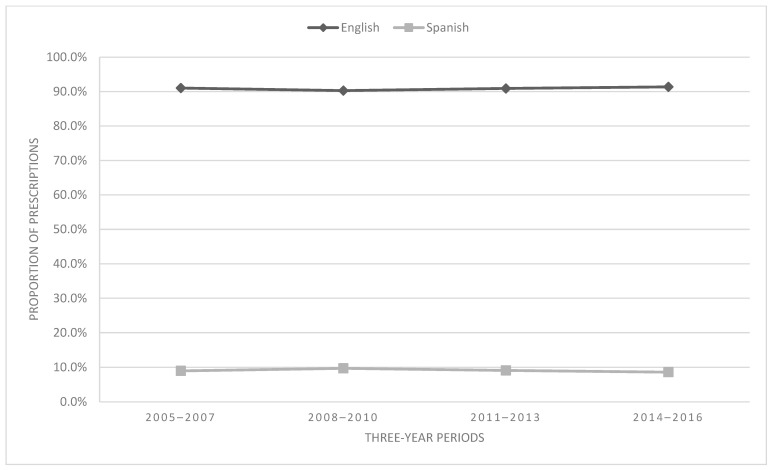
Proportion of prescriptions by language.

Most of the sample was English-speaking (90.8%); see Figure 6. Language and time were significantly associated [χ^2^ = 8.02, df = 3, *p* = 0.045, V = 0.01 (negligible effect size)]. English speakers demonstrated a proportional increase in opioid prescriptions of 0.4% from 2005–2007 to 2014–2016, while Spanish speakers demonstrated a 3.9% decrease (see Figure 6).

### 3.6. Prescribing Trends by Payer Type

**Figure 7:**
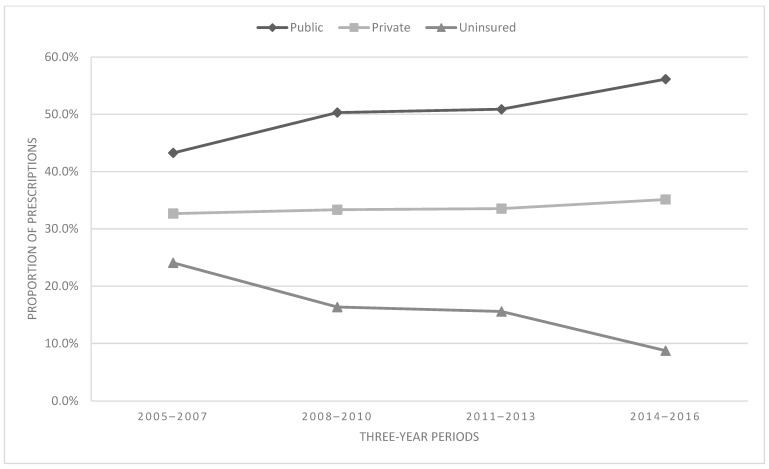
Proportion of prescriptions by payer type.

Figure 7 depicts prescribing trends by payer status. Most of the sample received some form of public health insurance plan (50.1%), followed by private insurance (33.6%) and uninsured (16.3%). There was a significant association between payer and time [χ^2^ = 819.64, df = 6, *p* < 0.001, V = 0.10 (small effect size)]. From 2005–2007 to 2013–2016, those on public health plans accounted for an increase of 29.8%, followed by private insurance (7.5%), while the uninsured demonstrated a decrease of 63.7%.

## 4. Discussion

The current study aimed to identify individual characteristics (e.g., age group, sex, ethnicity, race, language, and payer status) that were associated with differing trends over time in opioid prescribing patterns among pediatric patients before and after the peak of the prescription opioid epidemic using health record data from the primary children’s hospital system in a state highly impacted by opioid-related consequences. Within this sample, pediatric opioid prescriptions peaked from 2008 to 2010 and then returned to pre-peak levels by 2013–2016. Accompanying these macro trends in opioid prescriptions, we observed proportional changes in the number of prescriptions by age group, ethnicity, and payer, suggesting there were potentially interesting and as of yet unexplored changes in opioid prescriptions in subgroups during this sensitive time period.

Opioid prescribing trends over time were not consistent between subgroups; thus, opioid prescribing trends for the full sample of patients did not reflect prescribing trends among the subgroups. Within age groups, increases in opioid prescribing during the study were greatest for early childhood (+47.9%). Large increases in prescribing were also observed among school-age children (+22.2%) and adolescents (+10.2%). By contrast, the number of prescriptions for young adult patients decreased by 26.1% over this same period. Other notable findings were that opioid prescribing to non-Hispanic/Latine individuals increased over time, prescriptions to youth on public payer plans increased, and prescriptions to uninsured youth decreased. The current findings suggest that examining opioid prescribing trends to youth in aggregate may overlook potentially meaningful trends in prescribing from age and sociodemographic subgroups.

Few studies have examined opioid prescribing patterns among youth within distinct age groups [7,10]. Variations in prescribing trends between age groups observed in the current study contrast with results from Groenewald and colleagues, which found that opioid prescribing among age groups only increased in adults over age 18. The current findings align with Renney and colleagues’ outcomes, wherein overall dispensing rates to youth decreased for all ages (0–24 years); however, these researchers also found that the rates of extended-release/long-duration prescriptions increased dramatically for children aged 0–5 years and also increased slightly for youth aged 6–10. The large increase in opioid prescribing to school-age youth over time in the present sample is noteworthy and unique. Since the studies [7,10] that stratified by development groups utilized national data, it is possible that prescribing trends among age groups may differ uniquely by institution or geographical region.

Differences in opioid prescriptions/exposure by age are clinically important to consider because potential risks and side effects associated with prescribed opioids differ by age-related physiological characteristics (e.g., metabolic differences). For example, morphine, the opioid most commonly administered to children in a postoperative setting, is more slowly metabolized by infants younger than three months compared to infants and children older than three months [16]. Additionally, the nature of adverse effects of opioid exposure varies across development, raising differing considerations for patient and family education based on the child’s developmental level. For young children, the most frequent immediate adverse effects of opioid exposure are increased risk of mortality and accidental opioid poisoning. In fact, from 1999 to 2008, opioids were the most frequent medication to cause serious adverse outcomes, including injury and death, after accidental poisonings among children aged 0–5 years [17]. Conversely, adolescents and young adults who are prescribed opioids are more vulnerable to engaging in opioid misuse.

Another pertinent developmental consideration is that caregivers, namely, parents, are responsible for administering opioid prescriptions to children. Some data suggest that parents struggle to recognize the signs of opioid-related sedation in their child and, thus, may not withhold opioid doses as recommended, heightening the risk of accidental overdose [18]. Importantly, opioid dependence and withdrawal symptoms can be identified in children prescribed opioids after as little as seven days of use [19].

In the current study, opioid prescribing to non-Hispanic/non-Latine patients increased more than opioid prescribing to Hispanic/Latine patients, which aligns with the primary findings from Groenewald and colleagues’ longitudinal examination of opioid prescribing between pediatric racial and ethnic groups [7]. While our data did demonstrate significantly differing trends based on racial groups, the overall effect size was negligible.

Sex-related differences in opioid prescribing trends were negligible in this sample. This finding is similar to prior work that did not find sex-related differences in pediatric opioid prescribing practices [20]. Overall, males made up a greater proportion of overall prescriptions for opioids. This may be due to differences in prescribing practices but may also be due to other differences related to sex and predisposition to injuries and diseases that would require opioids for pain treatment. For example, boys (54%) account for more emergency visits in childhood compared to girls (46%) [21]. Regardless of the reason, higher prescribing to pediatric male patients is particularly noteworthy since adolescent males are more likely to engage in opioid misuse and have a higher prevalence of substance use disorders, although this gender gap is narrowing [22]. It is also important to note that women have a higher prevalence of chronic pain conditions, and these sex differences in prevalence rates emerge during adolescence [23]. However, despite the high prevalence of chronic pain conditions in females, pain remains undertreated among girls and young women. Indeed, gender bias is an emerging topic in pediatric medicine, and future studies should further explore potential biases in prescribing rates.

With respect to payer status, we observed that the proportion of opioid prescriptions increased for public insurance plans compared to a relative decrease in prescriptions to the uninsured. This may be reflective of treatment decisions made by providers but may also be due to systemic changes in the US payer system. Importantly related to this point, the Affordable Care Act (ACA) was enacted on 23 March 2010, and was implemented over the following years. Therefore, it may be possible that this shift in prescriptions is largely due to a shift in pediatric patients moving from uninsured to publicly insured status. Overall, the ACA reduced the number of uninsured by almost half (48.2 million to 28.2 million) between 2010 and 2016. Therefore, findings with respect to payer status must be interpreted cautiously and in light of the large changes in the structure of the US healthcare payment system.

### Limitations and Strengths

Medical records are not designed for research purposes and are therefore subject to limitations, including missing data, institutional changes in how data are collected over time, and a lack of flexibility for inclusivity in response options. To that end, while we noted some important differences in prescribing trends between racial and ethnic groups, it is imperative to interpret these results with some caution, as race was not reported by 27.1% of the sample, and ethnicity was not reported by 22.5%. Additionally, while the overall proportion of the sample identifying as White increased over time, other racial and ethnic groups remained relatively stable. This change may be driven by the decrease in individuals who did not report race, as well as a decrease in the group reported as “other.” Due to a lack of data on some groups and year-to-year variation (up and down), we decided to aggregate opioid prescriptions into three-year groups. This permitted analyzing groups that have low frequencies for any given year with a chi-square test. Also, we wanted to decrease the chance that a significant chi-squared test was detecting year-to-year variation and not trends of interest in the data. This decision involved a tradeoff, reducing the temporal fidelity of our results; that is, we were not able to detect changes at yearly timepoints but instead in three-year periods.

This study operates under the assumption that the demographics in the state and healthcare system remained consistent, as supported by US Census Bureau state population trend data, which indicate that trends for race and ethnicity remained relatively stable during the period of 2010–2019 [24]. It is a limitation that we could not determine whether changes in counts of prescriptions were related to groups of different demographics moving into or out of the University of New Mexico healthcare system. Future research should thoroughly test this assumption and consider higher levels of analysis (e.g., at the state or national level).

Additionally, these data lack denominator data, that is, the total number of pediatric visits where an opioid could have been given (e.g., pain- or injury-related visits). A primary limitation of collecting EHR data is the limited information obtained from each patient visit. While we observed changes in the trends of opioid prescriptions over the duration of the study period, we cannot say that there were changes in prescribing patterns during specific visits where opioids could be indicated. To answer this question, we would have needed additional types of data that we did not have access to.

We aggregated our results into three-year periods. In doing so, we gained confidence that significant results were due to observed trends and not simply due to year-to-year fluctuations. However, this also meant that precise analysis on a year-to-year basis was not possible and, therefore, is a limitation of the study.

Finally, another limitation of this study is that data collection ended in 2016. It is unknown how these trends may have changed since 2016, and therefore, these analyses should be complemented by future studies exploring how these trends have evolved since 2016. Despite this, a relative strength is the timeframe of this study (2005–2016), as it overlapped with the peak of opioid prescribing in the US and offers an intriguing window into current opioid concerns. Youth exposed to opioids during the study time period, especially those who were school age, are now adolescents and emerging young adults and may be individuals experiencing recent increases in opioid-related overdoses [25]. Thus, the current examination of pediatric opioid trends over time contributes to the growing body of literature trying to detail the evolution of the opioid epidemic among youth. To our knowledge, this is one of the few studies analyzing differences in opioid prescribing trends between different age groups in a pediatric patient population. It also contributes to the mounting data regarding sex, racial, and ethnic disparities among pediatric patients being treated for pain. The observed disparities in opioid prescribing based on these factors raise potential concerns about inequities in pain management.

## 5. Conclusions

The present study is a potential jumping-off point for several future investigations. Dissecting the variation in prescribing trends among sociodemographic groups can contribute to the creation of developmentally and socioculturally tailored patient education materials, as well as prescribing and pain management guidelines. While it is understood that opioid exposure in youth is associated with an increased risk of adverse outcomes, there remains a dearth of data examining whether opioid exposure during distinct childhood developmental periods is associated with differences in the risk of hazard. Prospective longitudinal work examining the short- and long-term benefits and consequences of opioid prescribing to pediatric populations is greatly needed and essential to understanding the individual factors that influence outcomes. Additionally, an examination of pediatric provider behavior and decision-making regarding opioid prescribing, in conjunction with the patient’s satisfaction with pain management services received, would provide valuable contextual information to the disparities observed in prescribing opioids to pediatric sociodemographic groups. In conclusion, we reiterate that just as age differences are an essential and established consideration in all other areas of pediatrics, physiological and psychosocial differences between age subgroups must be considered when evaluating the trends and effects of opioid prescriptions on pediatric populations.

## Figures and Tables

**Table 1:** Opioid prescriptions, proportion, and proportion of prescriptions by time period by patient characteristics.

	1. Opioid Prescriptions					2. Proportion of All Prescriptions					3. Proportion of Prescriptions for Time Period				
Age Group	2005–2007	2008–2010	2011–2013	2014–2016	Total Rx	2005–2007	2008–2010	2011–2013	2014–2016	Total %	2005–2007	2008–2010	2011–2013	2014–2016	% Change 2005–2007 to 2014–2016
0–5	1220	1999	1941	1630	6790	2.9%	4.8%	4.6%	3.9%	16.2%	12.8%	15.6%	17.6%	18.9%	47.9%
6–11	1329	2480	2156	1467	7432	3.2%	5.9%	5.1%	3.5%	17.7%	13.9%	19.3%	19.6%	17.0%	22.2%
12–17	2613	3362	2895	2601	11,471	6.2%	8.0%	6.9%	6.2%	27.3%	27.4%	26.2%	26.3%	30.2%	10.2%
18–21	4376	5006	4025	2920	16,327	10.4%	11.9%	9.6%	6.9%	38.9%	45.9%	39.0%	36.5%	33.9%	−26.1%
	9538	12,847	11,017	8618	42,020	22.7%	30.6%	26.2%	20.5%	100.0%	100.0%	100.0%	100.0%	100.0%	
**Sex**															
Male	5104	7165	6059	4765	23,093	12.1%	17.1%	14.4%	11.3%	55.0%	53.5%	55.8%	55.0%	55.3%	3.3%
Female	4434	5682	4958	3853	18,927	10.6%	13.5%	11.8%	9.2%	45.0%	46.5%	44.2%	45.0%	44.7%	−3.8%
	9538	12,847	11,017	8618	42,020	22.7%	30.6%	26.2%	20.5%	100.0%	100.0%	100.0%	100.0%	100.0%	
**Ethnicity**															
Hispanic/Latine	4419	6328	5727	4570	21,044	13.6%	19.4%	17.6%	14.0%	64.7%	73.9%	72.4%	58.8%	56.5%	−23.6%
Non-Hispanic/Latine	1557	2411	4005	3523	11,496	4.8%	7.4%	12.3%	10.8%	35.3%	26.1%	27.6%	41.2%	43.5%	67.1%
	5976	8739	9732	8093	32,540	18.4%	26.9%	29.9%	24.9%	100.0%	100.0%	100.0%	100.0%	100.0%	
**Language**															
English	8461	11,342	9669	7771	37,243	20.6%	27.7%	23.6%	19.0%	90.8%	91.0%	90.3%	90.9%	91.4%	0.4%
Spanish	834	1220	968	733	3755	2.0%	3.0%	2.4%	1.8%	9.2%	9.0%	9.7%	9.1%	8.6%	−3.9%
	9295	12,562	10,637	8504	40,998	22.7%	30.6%	25.9%	20.7%	100.0%	100.0%	100.0%	100.0%	100.0%	
**Race**															
White	3855	5475	5289	5366	19,985	14.6%	20.8%	20.1%	20.4%	75.9%	75.1%	74.6%	76.1%	77.6%	3.3%
AI/AN	920	1359	1178	1096	4553	3.5%	5.2%	4.5%	4.2%	17.3%	17.9%	18.5%	16.9%	15.8%	−11.6%
Black/AA	279	378	324	311	1292	1.1%	1.4%	1.2%	1.2%	4.9%	5.4%	5.1%	4.7%	4.5%	−17.3%
Asian	67	109	127	112	415	0.3%	0.4%	0.5%	0.4%	1.6%	1.3%	1.5%	1.8%	1.6%	24.1%
HN/PI	12	20	33	31	96	0.0%	0.1%	0.1%	0.1%	0.4%	0.2%	0.3%	0.5%	0.4%	91.7%
	5133	7341	6951	6916	26,341	19.5%	27.9%	26.4%	26.3%	100.0%	100.0%	100.0%	100.0%	100.0%	
**Payer**															
Public	4120	6463	5606	4838	21,027	9.8%	15.4%	13.3%	11.5%	50.1%	43.3%	50.3%	50.9%	56.1%	29.8%
Private	3111	4283	3695	3027	14,116	7.4%	10.2%	8.8%	7.2%	33.6%	32.7%	33.3%	33.5%	35.1%	7.5%
Uninsured	2293	2101	1716	753	6863	5.5%	5.0%	4.1%	1.8%	16.3%	24.1%	16.4%	15.6%	8.7%	−63.7%
	9524	12,847	11,017	8618	42,006	22.7%	30.6%	26.2%	20.5%	100.0%	100.0%	100.0%	100.0%	100.0%	

Note. AI/AN = American Indian or Alaska Native, AA = African American, HN = Hawaiian Native, and PI = Pacific Islander. This table describes (1.) the number of opioid prescriptions; (2.) the proportion of all opioid prescriptions (cells divided by total of all cells); and (3.) proportion of opioid prescriptions for each time period by category examined (each cell in a column divided by the column total).

## Data Availability

The primary data utilized in this study were accessed from the electronic health records of patients and therefore cannot be made publicly available.
